# Molecular mechanism of the treatment of lung adenocarcinoma by *Hedyotis Diffusa*: an integrative study with real-world clinical data and experimental validation

**DOI:** 10.3389/fphar.2024.1355531

**Published:** 2024-06-06

**Authors:** Sheng Wang, Na Yin, Yingyue Li, Zhaohang Ma, Wei Lin, Lihong Zhang, Yun Cui, Jianan Xia, Liang Geng

**Affiliations:** ^1^ The Affiliated Cancer Hospital of Zhengzhou University & Henan Cancer Hospital, Zhengzhou, China; ^2^ School of Medicine, Henan University of Chinese Medicine, Zhengzhou, China; ^3^ Medical Engineering Technology and Data Mining Institute, Zhengzhou University, Zhengzhou, China; ^4^ School of Computer Science and Technology, Beijing Jiaotong University, Beijing, China

**Keywords:** lung adenocarcinoma, Hedyotis diffusa, real world study, CTNNB1, complex network

## Abstract

**Background:**

With a variety of active ingredients, *Hedyotis Diffusa* (*H. diffusa*) can treat a variety of tumors. The purpose of our study is based on real-world data and experimental level, to double demonstrate the efficacy and possible molecular mechanism of *H. diffusa* in the treatment of lung adenocarcinom (LUAD).

**Methods:**

Phenotype-genotype and herbal-target associations were extracted from the SymMap database. Disease-gene associations were extracted from the MalaCards database. A molecular network-based correlation analysis was further conducted on the collection of genes associated with TCM and the collection of genes associated with diseases and symptoms. Then, the network separation S_AB_ metrics were applied to evaluate the network proximity relationship between TCM and symptoms. Finally, cell apoptosis experiment, Western blot, and Real-time PCR were used for biological experimental level validation analysis.

**Results:**

Included in the study were 85,437 electronic medical records (318 patients with LUAD). The proportion of prescriptions containing *H. diffusa* in the LUAD group was much higher than that in the non-LUAD group (*p* < 0.005). We counted the symptom relief of patients in the group and the group without the use of *H. diffusa*: except for symptoms such as fatigue, palpitations, and dizziness, the improvement rate of symptoms in the user group was higher than that in the non-use group. We selected the five most frequently occurring symptoms in the use group, namely, cough, expectoration, fatigue, chest tightness and wheezing. We combined the above five symptom genes into one group. The overlapping genes obtained were CTNNB1, STAT3, CASP8, and APC. The selection of CTNNB1 target for biological experiments showed that the proliferation rate of LUAD A549 cells in the drug intervention group was significantly lower than that in the control group, and it was concentration-dependent. *H. diffusa* can promote the apoptosis of A549 cells, and the apoptosis rate of the high-concentration drug group is significantly higher than that of the low-concentration drug group. The transcription and expression level of CTNNB1 gene in the drug intervention group were significantly decreased.

**Conclusion:**

*H. diffusa* inhibits the proliferation and promotes apoptosis of LUAD A549 cells, which may be related to the fact that *H. diffusa* can regulate the expression of CTNNB1.

## Introduction

Globally, the incidence and death rates of lung cancer are among the highest in the list of malignant tumors. Lung adenocarcinoma (LUAD) is a type of non-small cell lung cancer (NSCLC), which accounts for about 40%–55% of lung cancer patients (Herbst et al., 2018). LUAD tends to occur in female and male non-smokers, and is more common in China, and is characterized by brain, bone and liver metastases (Steinet al., 2019). LUAD usually has no obvious clinical symptoms in the early stage of the disease, and about 75% of patients are in the middle or late stage when they are found, with a low 5-year survival rate, which seriously affects the prognosis of patients (Wanget al., 2017). Although tyrosine kinase inhibitors and immunotherapy have brought some survival benefits to some patients with LUAD, the overall survival rate of patients is still low. Therefore, there is an urgent need to find safer and more effective drugs to treat LUAD.

Traditional Chinese medicine (TCM) has rich clinical experience and a large number of effective drugs in the treatment of tumors. Hedyotis diffusa (*H. diffusa*) is slightly bitter, sweet, cold in nature, belongs to the stomach, large intestine and small intestine meridians, and has the efficacy of clearing heat and removing toxins, inducing diuresis and clearing diuresis (Corraleset al., 2020). With a variety of active ingredients, *H. diffusa* can treat a variety of tumors such as digestive system tumors, lung cancer, prostate cancer and cervical cancer, and has anti-tumor effects such as inhibiting angiogenesis, inhibiting proliferation, and promoting apoptosis (Ma et al., 2014; Ye et al., 2019). Relevant studies have shown that the main antitumor components in *H. diffusa* are ursolic acid and oleanolic acid, and the antitumor mechanism of ursolic acid is to inhibit the growth of breast and colon cancer cell lines by triggering apoptosis, cell cycle arrest, and anti-metastatic and anti-angiogenic properties through various molecular targets and signaling pathways, and oleanolic acid is to induce cell apoptosis by regulating different signaling pathways and biological processes through multiple targets at the same time (He et al., 2018). Oleanolic acid, on the other hand, induces apoptosis by regulating different signaling pathways and biological processes through multiple targets, including intracellular calcium levels, NF-κB signaling pathway, Notch signaling pathway, JAK/STAT3 signaling pathway, and the expression of ribose polymerase (Chan et al., 2019). Based on the current research, there is still a lack of large samples of clinical data analysis and related mechanism studies on the treatment of LUAD with *H. diffusa*.

Symptom phenotypes (i.e., symptoms and signs), one of the main clinical manifestations of disease conditions, that could be obtained by human natural perception and cognition abilities, play a vital role for medical visiting, clinical diagnosis, and disease treatment. In recent years, researchers have developed a series of systems or network pharmacological strategies to detect the molecular mechanisms of TCM (Schroen, et al., 2015). A study extracted symptom co-occurrences from clinical textbooks to construct phenotype network of symptoms with clinical co-occurrence and incorporated high-quality symptom-gene associations and protein–protein interactions to explore the molecular network patterns of symptom phenotypes (Shu, et al., 2021). The work of Lu et al. showed that the integrated network analysis method could be used for identifying robust symptom clusters (SCs) and investigate the molecular mechanisms of these SCs, which would be valuable for symptom science and precision health (Lu, et al., 2020). By establishing a protein-protein interaction network framework, it was revealed that the general rule in TCM treatment selection is network distance (Gan, et al., 2023). By constructing a symptom-based human disease network, the authors analyzed the relationship between disease-symptom similarity and gene-protein interactions, and explored the relationship between the diversity of clinical manifestations of a single disease and its molecular mechanism (Zhou, et al., 2014). Taking advantage of GPU performance, the algorithm implementation of drug-symptom correlation analysis can be effectively accelerated (Tian, et al., 2020). Herb-Target Interaction Network (HTINet) approach obtained low-dimensional expression of nodes by constructing a heterogeneous network and performing network embedding, and used this expression to predict the relationship between Chinese herbal medicines and targets (Wang, et al., 2019). Using deep learning and heterogeneous network topology, a drug-target association prediction method that calculates the similarity between drug and target has been proposed (Zong, et al., 2019). A method using an iterative algorithm of three-layer heterogeneous networks to achieve drug repositioning is proposed (Wang, et al., 2014). A Network-based Random Walk with Restart (NRWRH) method is proposed to predict unknown drug-target interactions (Chen, et al., 2012).

**FIGURE 1:**
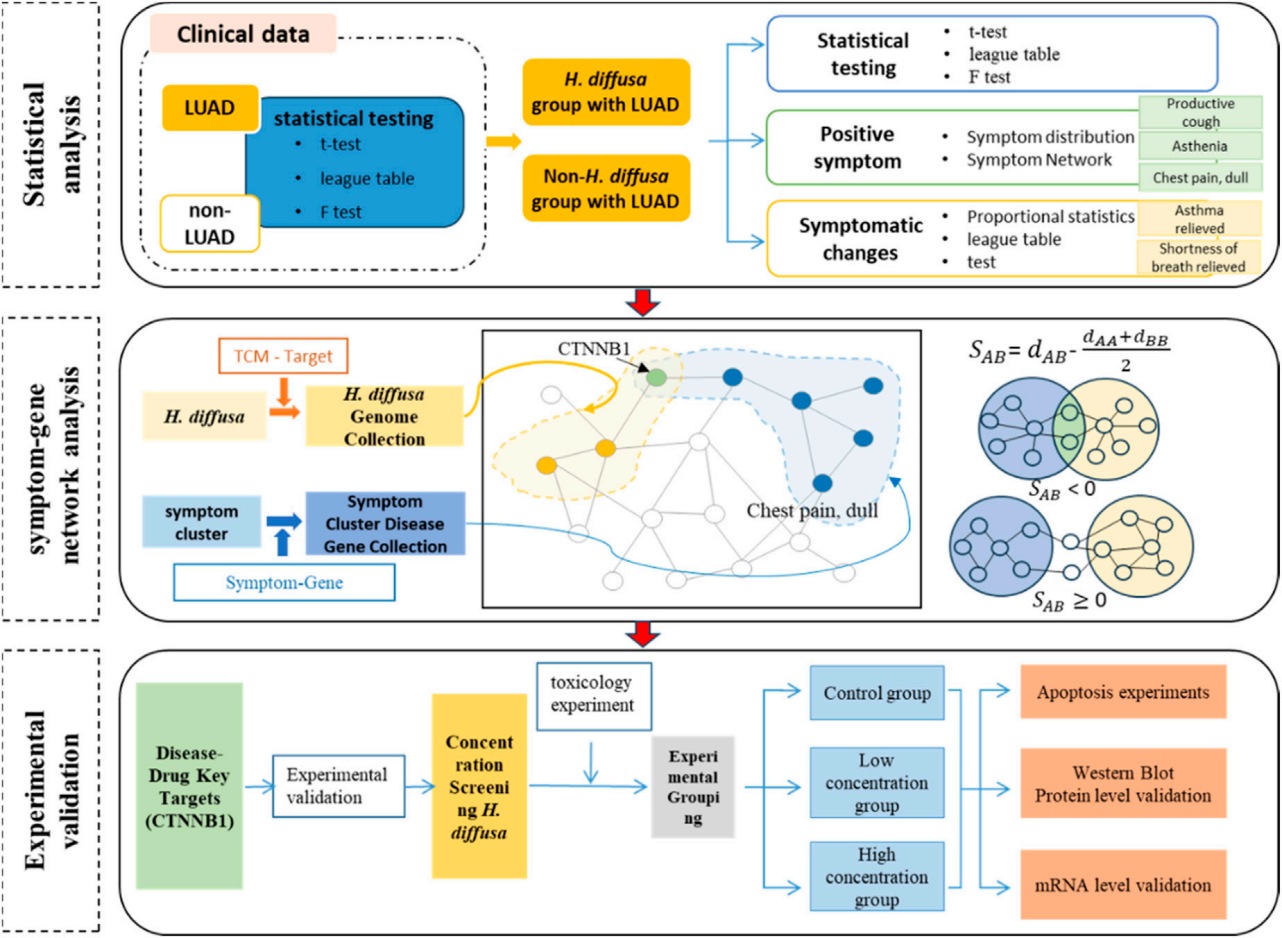
Schematic diagram of the technical process.

Based on real-world clinical data, this study reviewed and analyzed the application and symptom improvement of *H. diffusa* in LUAD, and performed relevant statistical analyses. In addition, the relationship network between symptom improvement and disease genes was also analyzed, and the gene target highly related to the symptom improvement of lung adenocarcinoma was finally screened out, and the molecular and cellular experiments were designed to validate the regulatory effect of *H. diffusa* on this target. The present study demonstrated the efficacy and possible molecular mechanism of Hedyotis diffusa in the treatment of lung cancer at both clinical and experimental levels, and provided the basis and new ideas for the study of the molecular mechanism of *H. diffusa* in the treatment of LUAD. The technical flowchart design we studied is shown below (Figure 1).

## Materials and methods

### Clinical data and preprocessing

The data of 85,437 inpatients were collected from the electronic medical records (EMRs) database of a provincial cancer hospital, including 318 patients with LUAD and 85,119 patients with non-LUAD, which included the patient’s age, gender, length of stay (LOS), admission records, discharge records, diagnosis information, and medical order information. The patients’ personal privacy was removed when the data was exported. Since patients’ admission records and discharge records were free text that cannot be directly used for statistical analysis, we used a clinical information extraction tool (SHU et al., 2020) to efficiently extract the biomedical entities (e.g., symptoms, diseases) from these records. Then, to normalize the various clinical term descriptions, we manually checked and standardized the terms “disease” and “herb” by referring to the 10th Revision of International Classification of Diseases (ICD-10) (Chabra S et al., 2019) and the Pharmacopoeia of the People’s Republic of China 2020 Revision (ChP 2020) (Chinese Pharmacopoeia Commission, 2020), respectively. In addition, The symptom data was standardized by two physicians in a back-to-back manner with reference to the Unified Medical Language System (UMLS) (Bodenreider et al., 2004).

### External data sources

The herbal target data and symptom-related gene data used in this article were downloaded from the SymMap (Wu et al., 2019) database (www.symmap.org). SymMap integrates traditional Chinese medicine (TCM) with modern medicine (MM) through both internal molecular mechanism and external symptom mapping, thus providing massive information on herbs/ingredients, targets, as well as the clinical symptoms and diseases they are used to treat for drug screening efforts. The SymMap database contains target sets of 618 herbal medicines, 832 TCM symptom genes, and a total of 20,965 targets/genes.

## Statistics analysis

To assess whether there is a correlation between two categorical variables, we used chi-square and t-tests on the processed data. The chi-square test evaluates the degree of association between variables (gender, symptoms) based on the difference between observed and expected frequencies. When performing the chi-square test, we first construct a contingency table to organize the observed frequency distributions of different categorical variables together. The expected frequency for each cell is then calculated, which is the frequency expected based on the assumed independence. Next, the differences between the observed and expected frequencies are calculated and these differences are squared and normalized. Finally, the chi-square statistic is obtained by summing the standardized differences. It can be used to compare the difference between the observed frequency and the expected frequency to determine whether the difference exceeds the range caused by randomness. The *t*-test is used to compare whether there are significant differences in the means (age, length of hospitalization) of two samples. When performing a *t*-test, we first calculate the mean and variance of each sample. Next, using these statistical indicators, we calculate the t-value, which represents the size of the difference between the two sample means relative to their standard errors.

### Drug-symptom molecular network analysis

In order to further study the mechanism behind the treatment of diseases by traditional Chinese medicine, a molecular network-based correlation analysis was further conducted on the collection of genes associated with traditional Chinese medicine and the collection of genes associated with diseases and symptoms. Network medicine leverages the human protein-protein interactome (PPI) to reveal disease and drug patterns. The PPI is a network consisting of nodes that are proteins that link to each other by physical (binding) interactions. Network medicine showed that disease-associated proteins tend to form locally clustered modules in the PPI, and shorter network distance between two disease modules is indicative of their comorbidity.

It is a method to measure the network relation between two node sets (e.g., target modules of herbs A and B) using the network distance d_AB_ and network separation S_AB_ metrics. The network distance d_AB_ is the average of network distances between all node pairs in two node sets. The network separation metric S_AB_ was designed to characterize disease-disease relation and drug-drug relation. S_AB_ compares the shortest distances between proteins d_AA_ and d_BB_ within each TCM and disease genome with d_AB_. For gene set A and gene set B participating in the protein network, the calculation formula of S_AB_ is as shown in the formula.

$$S_{AB}=d_{AB}-\frac{d_{AA}+d_{BB}}{2}$$

Among them, taking Chinese medicine (X) and symptoms (Y) as an example, the calculation of the shortest distance between the Chinese medicine target (x) and the symptom gene (y) is as shown in the formula.

$$d\left(X,Y\right)=\frac{1}{\left\|Y\right\|}\sum_{y\in Y}\min_{x\in X}d\left(x,y\right)$$

### Biological experiments

1) Cell culture and experimental treatments

A549 human lung cancer cells (Shenyang Wanlei Biology, Shenyang, China) were cultured in RMI1640 medium containing 20% FBS at 37°C and 5% CO^2^.

In experiments, A549 cells cultured to logarithmic growth stage were divided into six groups. Cells were treated with different doses of the *H*. *diffusa* (0, 50, 75, 100, 125, 150 μg/mL) for 48 h.

2) CCK-8 assay

After treatment of cells, the culture medium was removed and replaced with 100 μL fresh medium in each well. Next, 10 μL CCK-8 was added and cells were incubated at 37°C for 1 h. The OD value at 450 nm was measured on a microplate analyzer, and data analysis was performed.

3) Flow cytometry

Cell apoptosis was detected by staining with Annexin V-FITC/PI. Cells were collected by centrifugation and the supernatant was discarded; cells were washed twice with PBS and 500 μL binding buffer was added. Next, 5 μL Annexin V-FITC and 10 μL propidium iodide were added and cells were incubated at room temperature in the dark for 5–15 min. Analysis by flow cytometry was performed.

4) Western blot

Cells were lysed in RIPA buffer containing PMSF, phosphatase inhibitor and protease inhibitor (at 100:1:1:1). Protein quantification was performed using a BCA protein quantification kit. Lysates were boiled in 5× loading buffer and separated on 10% SDS-PAGE gels at 80 V for 30 min and 120 V for 50 min, followed by transfer to a PVDF membrane. Membranes were blocked in 5% skim milk-TBST at room temperature for 2 h. The TBST membrane was washed three times, 8 min each time, and 4°C incubated with primary antibody overnight. Primary antibodies were anti-EGFR, KDR, MAPK3, PTPN11 and CTNNB1 (1:1,000; KDR, MAPK3 and PTPN11) and anti-GAPDH (1:1,000). The membrane was washed three times with TBST for 8 min each and incubated with secondary antibody at room temperature for 2 h. Secondary antibodies included HRP-labeled sheep anti-rabbit IgG (1:10,000) and HRP-labeled sheep anti-mouse IgG (1:10,000). The blots were processed using an ECL kit and the bands were analyzed using a gel image processing system (Gel-Pro-Analyzer software).

(5) Real-time PCR

RNA was extracted from cells by Trizol-chloroform-isopentyl alcohol method (Thermo, United States), and cDNA was synthesized by reverse transcription kit (Servicebio, Wuhan, China). The relative expression level of DKC1 was detected by real-time fluorescence quantitative PCR. Primer sequences are shown in Table 1. Reaction system: 2×qPCR Mix 12.5 μL, 2.5 μM primer 0.5 μL, reverse transcription product 2.0 μL, ddH2O 4.0 μL. PCR amplification: pre-denaturation: 95°C, 10 min; Cycle (40 times): 95°C, 15 s, 62°C, 30 s, 72°C, 30 s; Melting curve: 60°C→95°C, 0.5°C every 15 s. With β-actin as internal reference, mRNA relative expression was calculated by 2^−△△Ct^ method.

**TABLE 1:** Primer sequence list.

Name	Primer sequence
homo KDR F	AAT​AAT​CAG​AGT​GGC​AGT​G
homo KDR R	ACATAAATGACCGAGGC
homo MAPK3 F	GGGAGGTGGAGATGGTG
homo MAPK3 R	GCT​GGC​AGT​AGG​TCT​GAT​GT
homo PTPN11 F	AGA​GGA​GTT​GAT​GGC​AGT​T
homo PTPN11 R	TTC​TGA​ATC​TTG​ATG​TGG​G
homo β-actin F	GGCACCCAGCACAATGAA
homo β-actin R	TAGAAGCATTTGCGGTGG

## Results

### Analysis of the role of *H. diffusa* in patients with LUAD

Firstly, based on the data as shown in Table 2, the proportion of LUAD patients using prescriptions containing *H. diffusa* is 39%, much higher than that of the non-LUAD group (*p* < 0.005).

**TABLE 2:** Statistics on LUAD patients and users of *H. diffusa*.

	LUAD (Proportion)	Non-LUAD (Proportion)	Total
Used group	124**(39%)**	6,796**(8%)**	6,920
Unused group	194**(61%)**	78,323**(92%)**	78,517
Total	318	85,119	85,437

Secondly, based on the LUAD patient group, the demographic statistics between the group using *H. diffusa* and the group not using it were calculated. We extracted patient symptoms from admission and discharge medical records through manual annotation and a clinical information extraction tool (Human-machine Cooperative Phenotypic Spectrum Annotation System, HCPSAS; www.tcmai.org). Table 3 presents the statistics comparison between *H. diffusa* used group and unused group, only symptom cough and sleep disturbances show statistical difference.

**TABLE 3:** Hospitalization information statistics of used group and unused group.

	Used group	Unused group	*p*-value
AGE	66.22	66.98	*p* = 0.279
SEX (Male/Female)	83/41	121/73	*p* = 0.408
InhosTime	14.78	14.99	*p* = 0.429
Non-productive cough (admission)	76/48	92/102	*p* = 0.016
White-colored sputum (admission)	40/84	47/147	*p* = 0.117
Productive cough (admission)	39/85	52/142	*p* = 0.371
Asthenia (admission)	38/86	75/119	*p* = 0.145
Chest pain, dull (admission)	36/88	69/125	*p* = 0.227
Asthma (admission)	32/92	50/144	*p* = 0.995
Decreased appetite (admission)	29/95	46/148	*p* = 0.947
Sleep disturbances (admission)	23/101	76/118	*p* = 0.000
Viscid sputum (admission)	20/104	35/159	*p* = 0.660
Scanty sputum (admission)	18/106	20/174	*p* = 0.259

Finally, statistical analysis was performed on symptoms that were significantly relieved among discharged cases. We counted the relieved symptoms of patients in the used group and unused group respectively, and sorted them in descending order according to the number of symptoms in the used group, the number in brackets is the total number of symptoms in this group. Except for symptoms of Asthenia, Heart pounding, and Dizziness, the improvement rate of symptoms in the used group was higher than that in the unused group, as shown in Table 4.

**TABLE 4:** Statistics on symptom improvement in the used group and unused group.

Relieved symptom	Used group		Unused group		Improvement ratio comparison	
Non-productive cough relieved	25	(76)	26	(92)	**32.89%**	28.26%
Chest pain, dull relieved	22	(36)	24	(69)	**61.11%**	34.78%
Asthma relieved	19	(32)	17	(50)	**59.38%**	34.00%
Productive cough relieved	17	(39)	18	(52)	**43.59%**	34.62%
Asthenia relieved	6	(38)	23	(75)	15.79%	**30.67%**
Shortness of breath relieved	4	(9)	4	(11)	**44.44%**	36.36%
Breath-holding spells relieved	3	(4)	2	(8)	**75.00%**	25.00%
Headache, cephalalgia relieved	3	(8)		(5)	**37.50%**	0.00%
Heart pounding relieved	2	(11)	6	(24)	18.18%	**25.00%**
Dizziness relieved	2	(15)	5	(17)	13.33%	**29.41%**
Dyspnea relieved	2	(3)	3	(5)	**66.67%**	60.00%
Xerostomia relieved	2	(5)	3	(9)	**40.00%**	33.33%

Bold values represents the highest proportion.

### Herb-symptom network analysis

First, based on the symptom statistics of admitted patients, we selected the five most frequently occurring symptoms in used group, which are Non-productive cough, Productive cough, Asthenia, Chest pain, dull and Asthma. For these symptoms, we perform exact matching or approximate matching in the symptom database and obtain the number of genes shown in Table 5.

**TABLE 5:** Target and gene set information for specific herbs and symptoms.

	Herb/Symptom name	Target/Gene	Number of gene
Herb	*H. Diffusa (Bai hua she cao)*	**CTNNB1**, APC, CASP1, GCG, ••••••, CTSD, EGF	353
Symptom	Non-productive cough	STAT3, IKZF1, BLNK, WWOX, TCF3, ••••••, MARS, VCP, DLEC1, IGHM, TSC1	39
	Productive cough	STAT3, BLNK, GBA, IGLL1, CD79A, ••••••, IGHM, MARS, GAS8, EPOR, VCP	39
	Asthenia	MRPL45, MRPL51, CRAT	3
	Chest pain, dull	**CTNNB1**, APC, PTPN22, MPL, LOX, ••••••, WAS, MFAP5, SMAD3, CALR, DLEC1	44
	Asthma	CASP8, ERCC2, PEPD, FLG, UFD1, ••••••, IDS, PGM3, NEK9, TBX1, ADA	45

Based on the above symptom gene set and herbal medicine target set, we combined the above five symptom genes into one group, and put the total symptom gene set and herbal medicine target set into the gene association analysis platform respectively. The overlapping genes obtained are CTNNB1, STAT3, CASP8 and APC. Previous studies have shown that the CTNNB1 gene is related to the occurrence and development of tumors, but the mechanism of LUAD has not been carried out. Next, we used biological experiments to further verify and study the mechanism.

**FIGURE 2:**
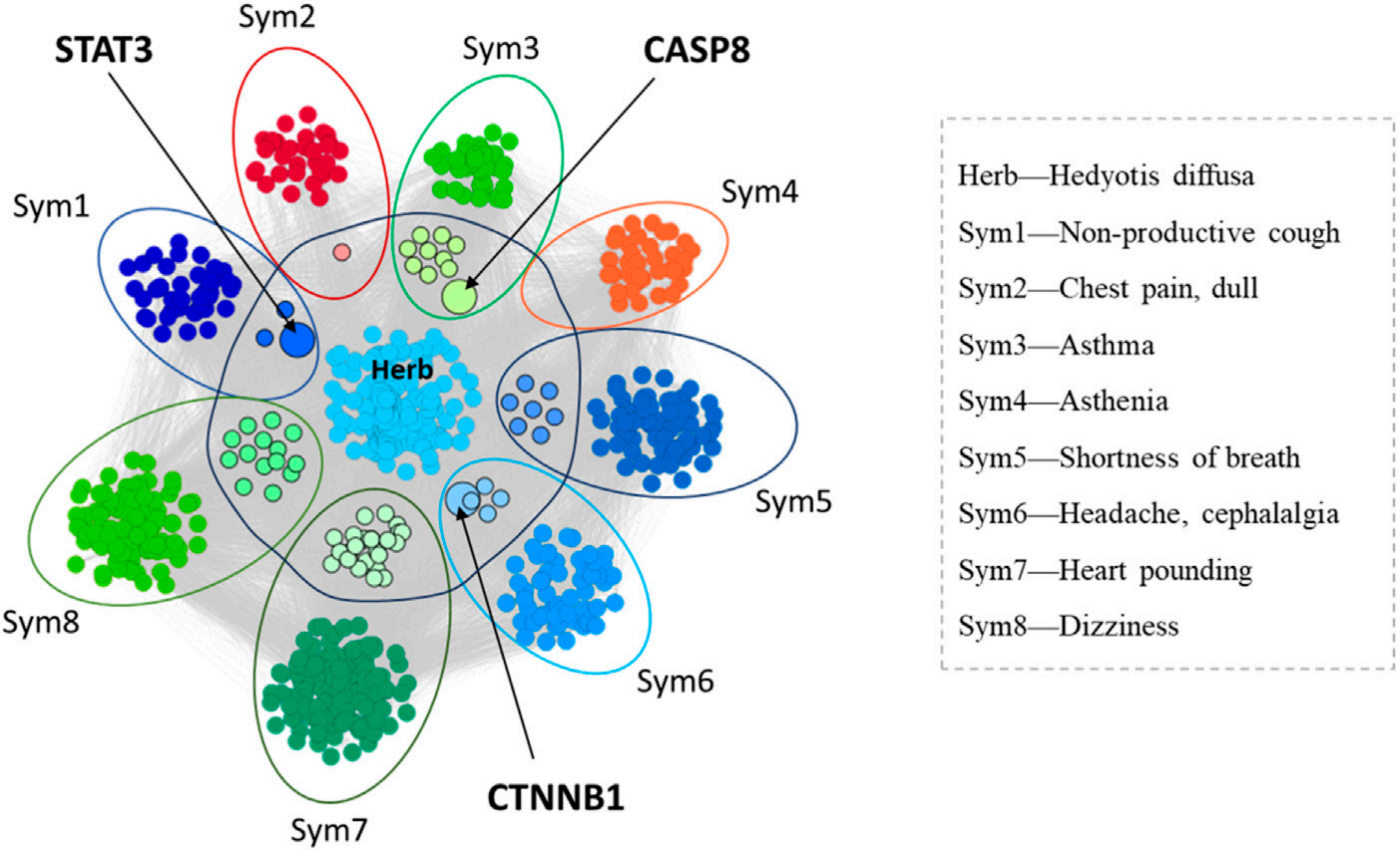
Illustration diagram of S_AB_ analysis between Hedyotis diffusa and relieved symptom.

Then, the ten symptoms with the highest degree of improvement in the used group were selected for analysis, and their S_AB_ values for the Hedyotis diffusa target set and the gene set of the ten symptoms were calculated respectively. The calculation results are shown in Table 6 and the illustration diagram is shown in Figure 2. For S_AB_ < 0, it means that the targets (genes) of the two traditional Chinese medicines and symptoms are located in the same network area. The smaller the value, the stronger the correlation. Therefore, when evaluating the correlation between traditional Chinese medicine and symptom genome, if the value of S_AB_ is negative, its significance is more important, which means that there is a closer correlation between traditional Chinese medicine and symptom genome. From the results table, it can be seen that the S_AB_ values of Hedyotis diffusa and symptoms such as Non-productive cough, Shortness of breath, Breath-holding spells, Headache, cephalalgia and Xerostomia are all less than 0. Combined with the statistical results of the improvement ratio comparison in the previous section, it is verified that Hedyotis diffusa has a positive effect on the above symptoms.

**TABLE 6:** Sab value between specific herbs and symptoms.

Herbal name	Relieved symptom	Sab
Hedyotis diffusa	Non-productive cough relieved	−0.23318
Hedyotis diffusa	Chest pain, dull relieved	0.27559
Hedyotis diffusa	Asthma relieved	0.00877
Hedyotis diffusa	Productive cough relieved	-
Hedyotis diffusa	Asthenia relieved	0.41871
Hedyotis diffusa	Shortness of breath relieved	−0.24698
Hedyotis diffusa	Breath-holding spells relieved	−0.24697
Hedyotis diffusa	Headache, cephalalgia relieved	−0.20866
Hedyotis diffusa	Heart pounding relieved	0.06504
Hedyotis diffusa	Dizziness relieved	0.06700
Hedyotis diffusa	Dyspnea relieved	−0.24697
Hedyotis diffusa	Xerostomia relieved	-

## Experimentation

### Toxicological effects of the *H*. *diffusa* on lung cancer cells

We treated LUAD cells with different concentrations of *H*. *diffusa* (0 μM/mL, 50 μM/mL, 75 μM/mL, 100 μM/mL, 125 μM/mL, 150 μM/mL). The results are shown in Table 7. The inhibitory effects of *H*. *diffusa* on the activity of A549 LUAD cells were (14.99 ± 2.31) %, (44.83 ± 3.42) %, (62.39 ± 5.23) %, (78.46 ± 5.56) %, and (90.67 ± 6.21) %, respectively, indicating that inhibition occurred in a concentration-dependent manner. The IC50 concentration of A549 cells was 79.65 ± 5.52 μM/mL; 60 μM/mL was selected as low concentration, and 125 μM/mL was selected as high concentration to treat A549 cells.

**TABLE 7:** Inhibitory effects of the *H. diffusa* at different concentrations on A549 LUAD cells.

A549	Absorbance (450 nm)	inhibition ratio (%)	*p*
A: Control Group	0.799 ± 0.018	0	
B: 50 μg/mL Group	0.678 ± 0.026	14.99 ± 2.31	0.0392
C: 75 μg/mL Group	0.440 ± 0.029	44.83 ± 3.42	0.0011
D: 100 μg/mL Group	0.300 ± 0.020	62.39 ± 5.23	0.0002
E: 125 μg/mL Group	0.172 ± 0.013	78.46 ± 5.56	<0.0001
F: 150 μg/mL Group	0.074 ± 0.007	90.67 ± 6.21	<0.0001

### Effects of *H*. *diffusa* on apoptosis of lung cancer cells

**FIGURE 3:**
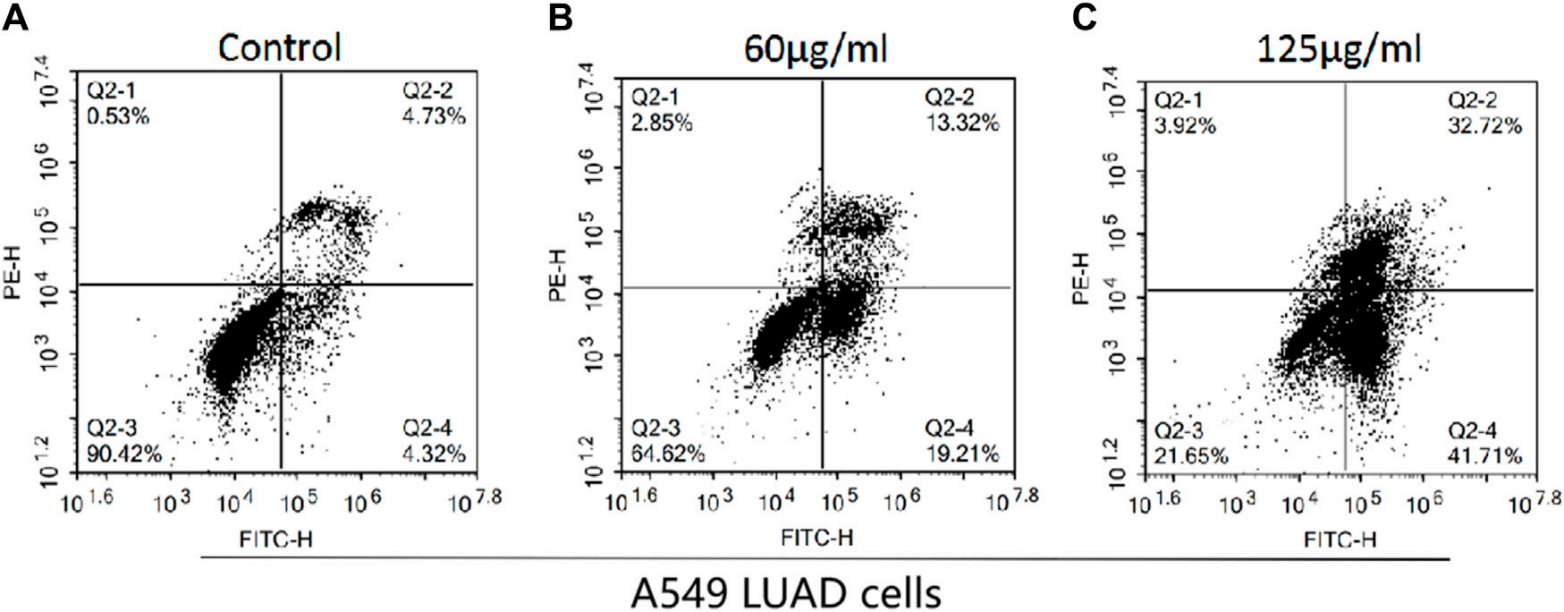
Effects of the *H*.*diffusa* on the apoptosis of A549 LUAD cells. **(A)**, Control group; **(B)**, Low concentration group; **(C)**, High concentration group.

Flow cytometry was used to detect the effects of *H*. *diffusa* components on A549 LUAD cell apoptosis, and the results are shown in Figure 3. The apoptosis rates of the control group, the low concentration group and the high concentration group were 9.05%, 32.53%, and 74.43%, respectively. These results indicate that *H*. *diffusa* promote the apoptosis of lung cancer cells.

### Regulatory effects of *H.diffusa*on on CTNNB1 in lung cancer cells

**FIGURE 4:**
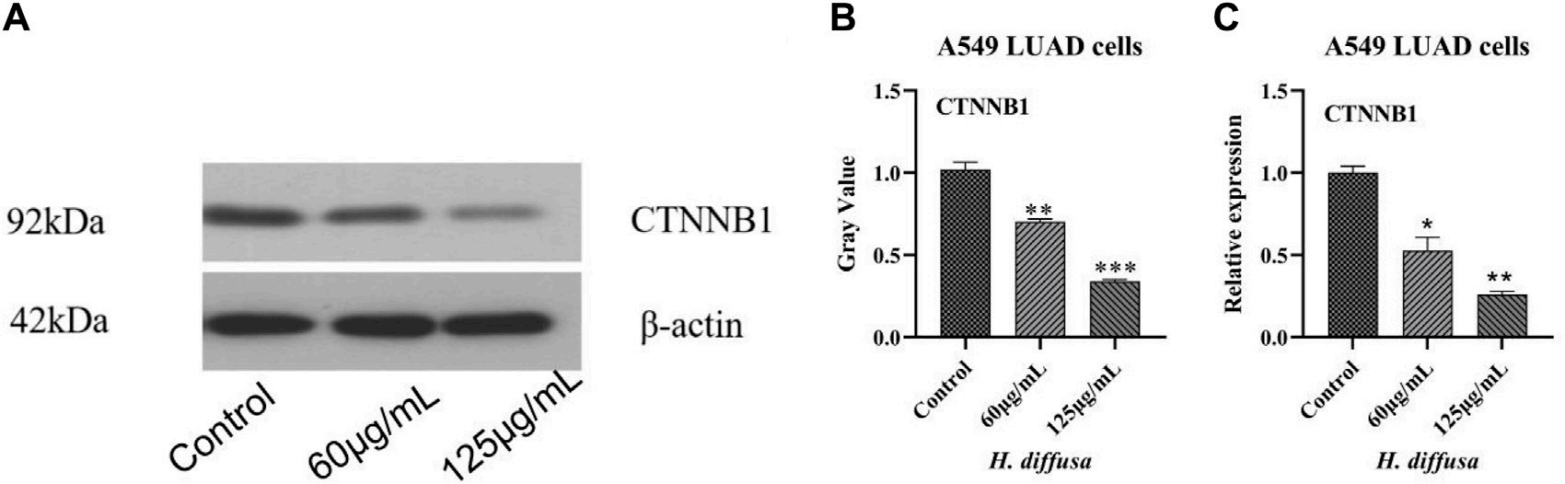
Effects of *H.diffusa* on CTNNB1 in A549 LUAD cells. **(A)**, Western blot analysis. **(B)**, Gray analysis. **(C)**, mRNA levels analysis. *p < 0.05, **p < 0.01,***p < 0.001, compared to the control group.

Western blot was used to detect the effects of *H*.*diffusa* on key proteins of LUAD, and the results are shown in Figures 4A,B. The expression levels of CTNNB1 proteins in cells treated with the *H*.*diffusa* were significantly lower than those in the control group (*p* < 0.05). In addition, the protein expression level of the high concentration group was significantly lower than that of the low concentration group (*p* < 0.05).

Further to the above CTNNB1 target detect the mRNA level of analysis, the results as shown in Figure 4C, mRNA expression level and protein level consistent. The mRNA expression levels of CTNNB1 in cells treated with the *H.diffusa* were significantly lower than those in the control group (*p* < 0.05). Inaddition, them mRNA expression level of the high concentration group was significantly lower than that of the low concentration group (*p* < 0.05).

## Discussion

LUAD is the most common subgroup of NSCLC with aggressive and metastatic potential, leading to drug resistance and treatment failure (Wang et al., 2009; Travis et al., 2015). Despite advances in the detection and treatment of NSCLC, unfortunately, the prognosis remains poor when the disease is detected in late clinical stages, and some patients are still plagued by rapid disease recurrence and progression, thus resulting in a 5−year survival rate of only about 15% (Erridge et al., 2007). TCM participation is an important means in the treatment of LUAD. Due to the characteristics of syndrome differentiation and treatment, combined with surgery, chemotherapy and targeted therapy, TCM plays an anti-tumor role from the two aspects of self-healing ability and disease resistance. Recently, an increasing number of studies (Wu et al., 2022; Huang et al., 2022; Qian et al., 2019) showed that the herb has some antitumor effects, including in the treatment of LUAD. It is a kind of Chinese herbal herb family, which is a famous Chinese herbal medicine with thousands of years of clinical practice history. It is an important component of various anticancer formulations; it was reported to inhibit tumor cell proliferation and metastasis and reduce side effects after chemotherapy (Song et al., 2019). Early pharmacological studies confirmed that the herb has medicinal properties such as anti-tumor, anti-inflammatory, immunoregulatory, antioxidant and other biological activities (Shen et al., 2016). The current studies on the mechanism of lung adenocarcinoma focus on the theoretical stage of network pharmacology or only *in vitro* experimental stage, and the analysis of real world clinical data has not been reported. Based on this, based on real-world clinical data, our study analyzed the key targets of LUAD from the perspective of drug improvement of symptoms, and used biological experiments to verify them, so as to provide certain theoretical basis and support for the mechanism of LUAD in TCM.

*H. diffusa* is an important component of TCM clinical anticancer prescription (Han et al., 2020; Wazir et al., 2021; Zhang et al., 2021), and the results of this study showed that it is used significantly more frequently in patients with LUAD than in patients with other diseases (*p* < 0.05). A literature study on the treatment of NSCLC (Qi et al., 2021) showed that the herb is one of the more frequent Chinese medicines in prescription. At the same time, many studies in Taiwan have shown that it is also one of the most frequently prescribed TCM (Hung et al., 2017; Kuo et al., 2018; Ting et al., 2017; Wang et al., 2021) in the treatment of other cancers (such as liver cancer, gastric cancer, nasopharyngeal cancer and pancreatic cancer). An experimental study (Huang et al., 2022) showed that the *H. diffusa* injection significantly reduced the survival of lung adenocarcinoma cells *in vitro*, inhibited the growth of BALB/c nude mice *in vivo*, and induced iron death of VDAC 2/3 regulation by Bax by inhibiting Bcl-2 gene. In addition, the results of this study showed a significant improvement in the clinical symptoms (such as cough, chest tightness, wheezing, sputum, shortness of breath, headache, dry mouth), and improved the quality of life.

Most of the previous studies have focused on the relationship between TCM and disease, for example, TCM diagnosis and herbal therapy are based on the symptom phenotype rather than disease diagnosis. Thus, our study builds on symptoms rather than disease. By focusing on symptoms rather than disease, our approach is consistent with the practice of TCM in diagnosing and treating patients based on the symptom phenotype. The study by Gan et al. (Gan et al., 2023) found that proteins associated with symptoms tend to cluster in a local PPI module, and the network proximity between herbal targets and symptom modules indicates the effectiveness of herbs in treating symptoms. Based on the above improvement symptom gene set and the TCM target set, we combined the above five symptom genes into one group, and put the total symptom gene set and the TCM target set into the gene association analysis platform respectively. The overlapping genes obtained were CTNNB1, STAT3, CASP8, and APC. We picked out the highest weighted target CTNNB1 to unfold the following validation. WANG et al. (Wang et al., 2017) used network pharmacology to analyze the potential targets of *H. diffusa-Astragalus membranaceus* for the treatment of colorectal cancer, and found that the core target also contained CTNNB1. Zhou et al. (Zhou et al., 2020) included 564 patients with LUAD in the study and found a poor prognosis in patients with primary lung adenocarcinoma with CTNNB1 mutation. *In vitro*, we combined the *H. diffusa* with lung adenocarcinoma A549 cells, and the apoptosis experiment showed that the higher concentration of drug groups promoted apoptosis more than the lower concentration. Furthermore, WANG et al. (Wang et al., 2022) showed that *H. diffusa* significantly reduced the survival rate of lung adenocarcinoma cells *in vitro* and significantly inhibited the cell adhesion, invasion and migration of lung adenocarcinoma A549 cells. We further verified the effect of *H. diffusa* on CTNNB1 at the mRNA and protein levels, and found that compared with the control group, the drug group significantly inhibited the mRNA and protein levels of CTNNB1 gene, and the inhibitory effect of the high drug group was more obvious than that of the low concentration of the drug group. It may speculate that it may promote apoptosis of lung cancer A549 cells by inhibiting the CTNNB1 gene. In addition, the shortcomings of our study compared to the study by Peng et al. are that we did not consider prescriptions, but rather looked at the effects of individual drugs (Wang et al., 2022; Peng et al., 2023).

In conclusion, our research based on real world data from clinical LUAD symptoms changes and the relationship between *H. diffusa*, using network algorithm for key targets, and through the experiment verified the key target, the whole argument method is rigorous, can for clinical treatment of lung adenocarcinoma provide certain theoretical support.

## Data Availability

The original contributions presented in the study are included in the article/Supplementary Material, further inquiries can be directed to the corresponding authors.
