# Supplementary figures and images for: Molecular mechanism of the treatment of lung adenocarcinoma by Hedyotis Diffusa: an integrative study with real-world clinical data and experimental validation
Source: Front Pharmacol. 2024 Jun 6;15:1355531. doi: 10.3389/fphar.2024.1355531 (PMC11187350; doi:10.3389/fphar.2024.1355531)

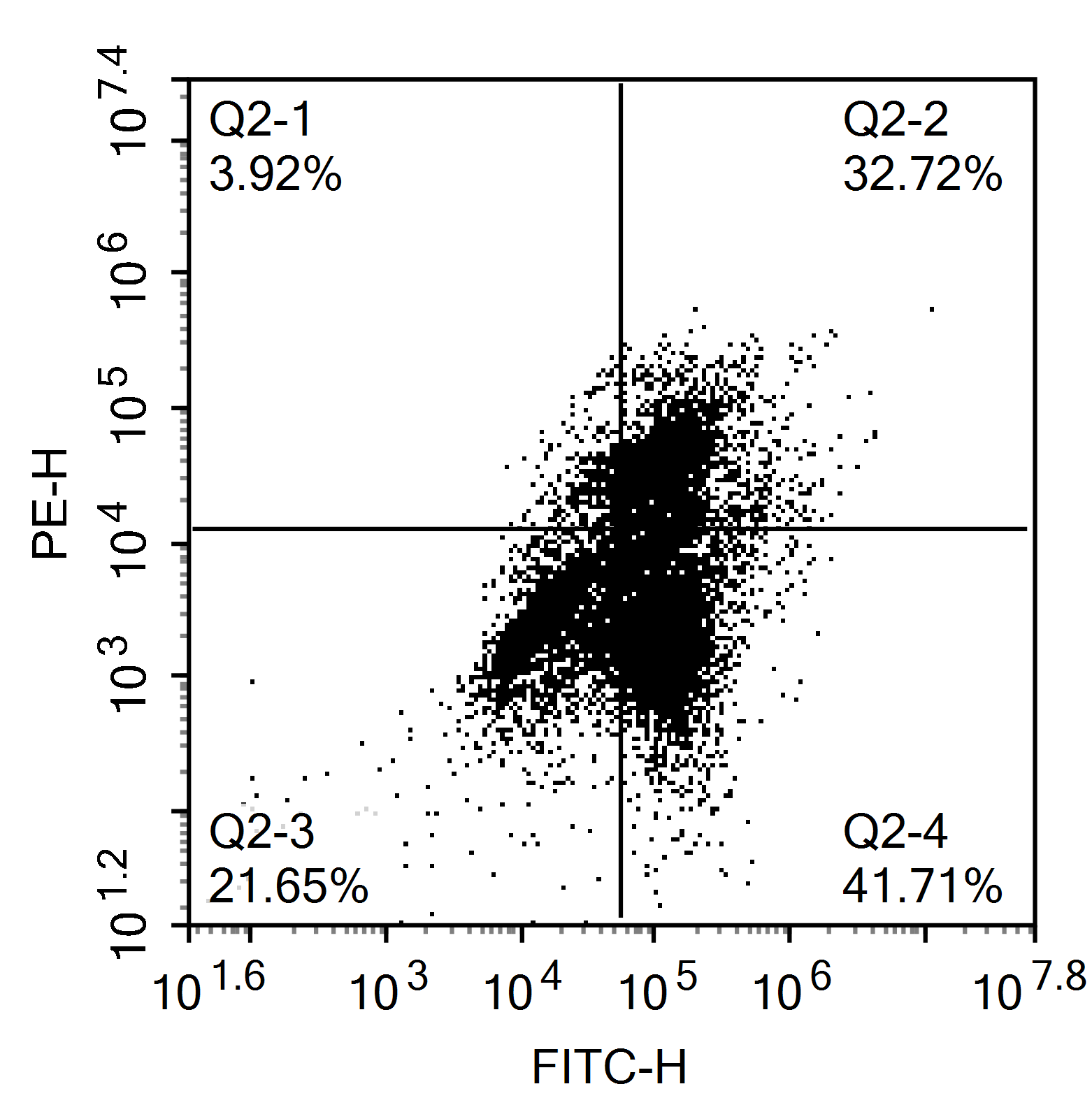

Supplement: Supplementary file 2 [file DataSheet2.ZIP › Experimental data/Apoptosis experiments--High-dose group.tiff]

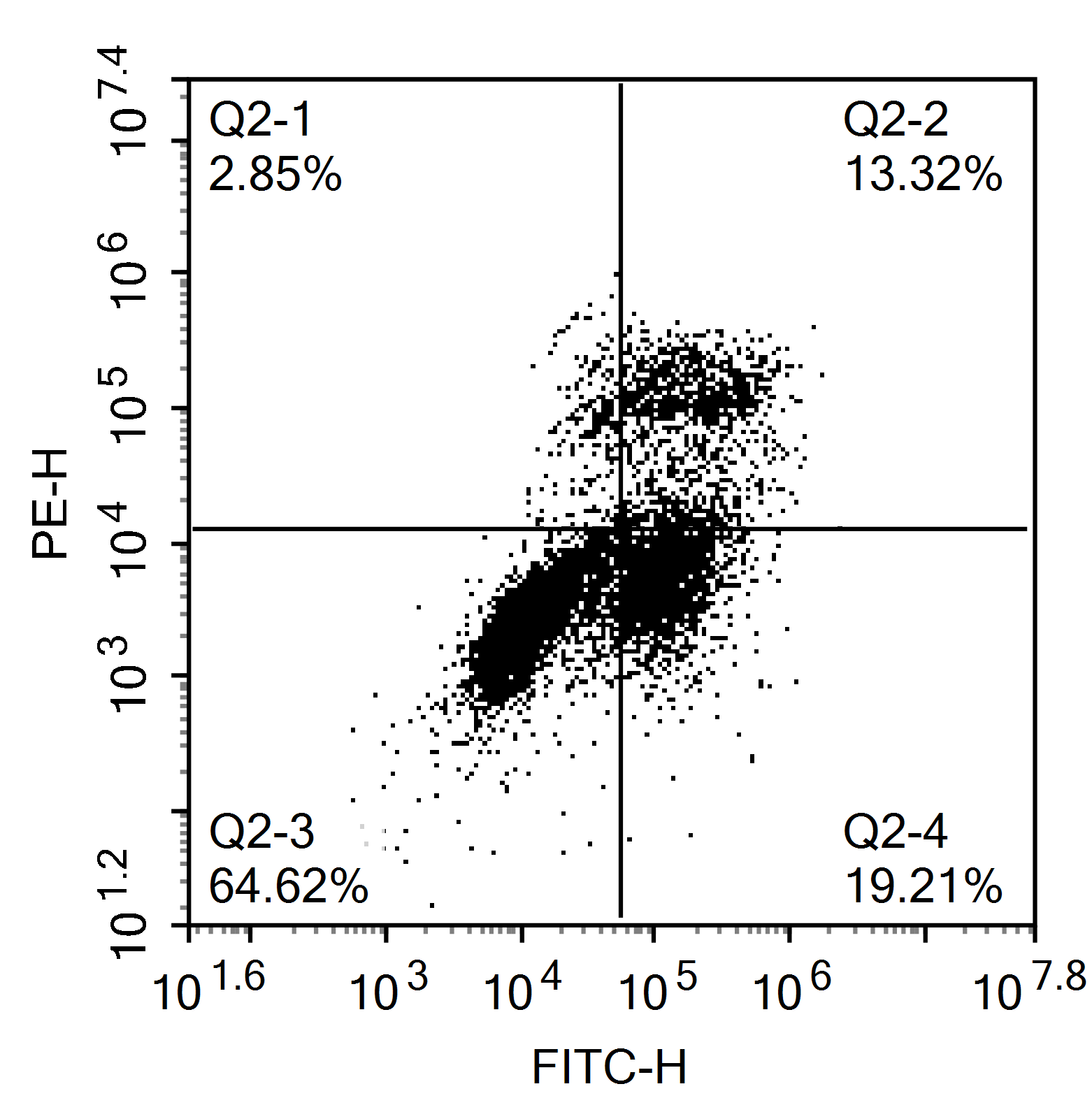

Supplement: Supplementary file 2 [file DataSheet2.ZIP › Experimental data/Apoptosis experiments--Low-dose group.tiff]

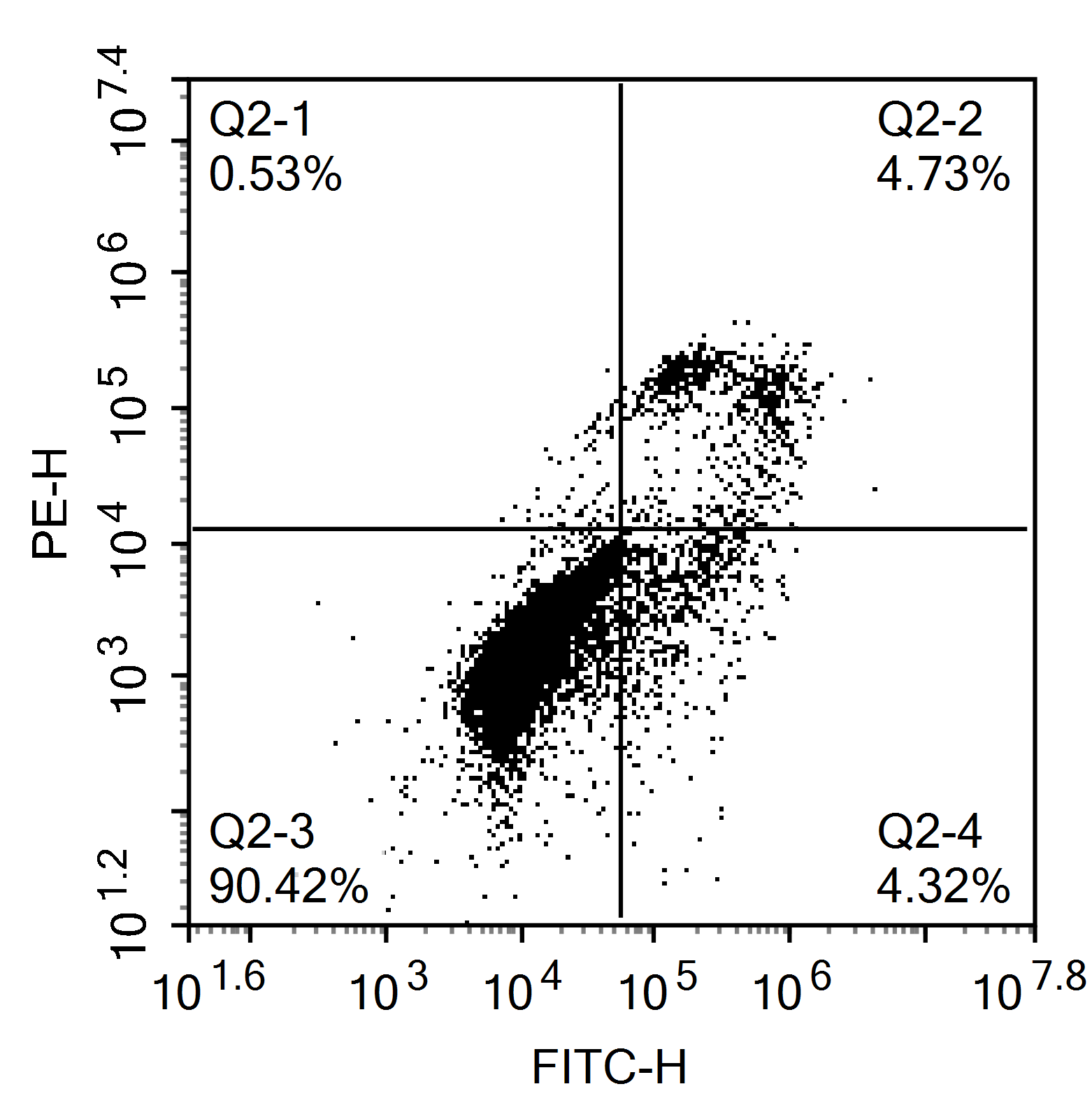

Supplement: Supplementary file 2 [file DataSheet2.ZIP › Experimental data/Apoptosis experiments-Normal group.tiff]

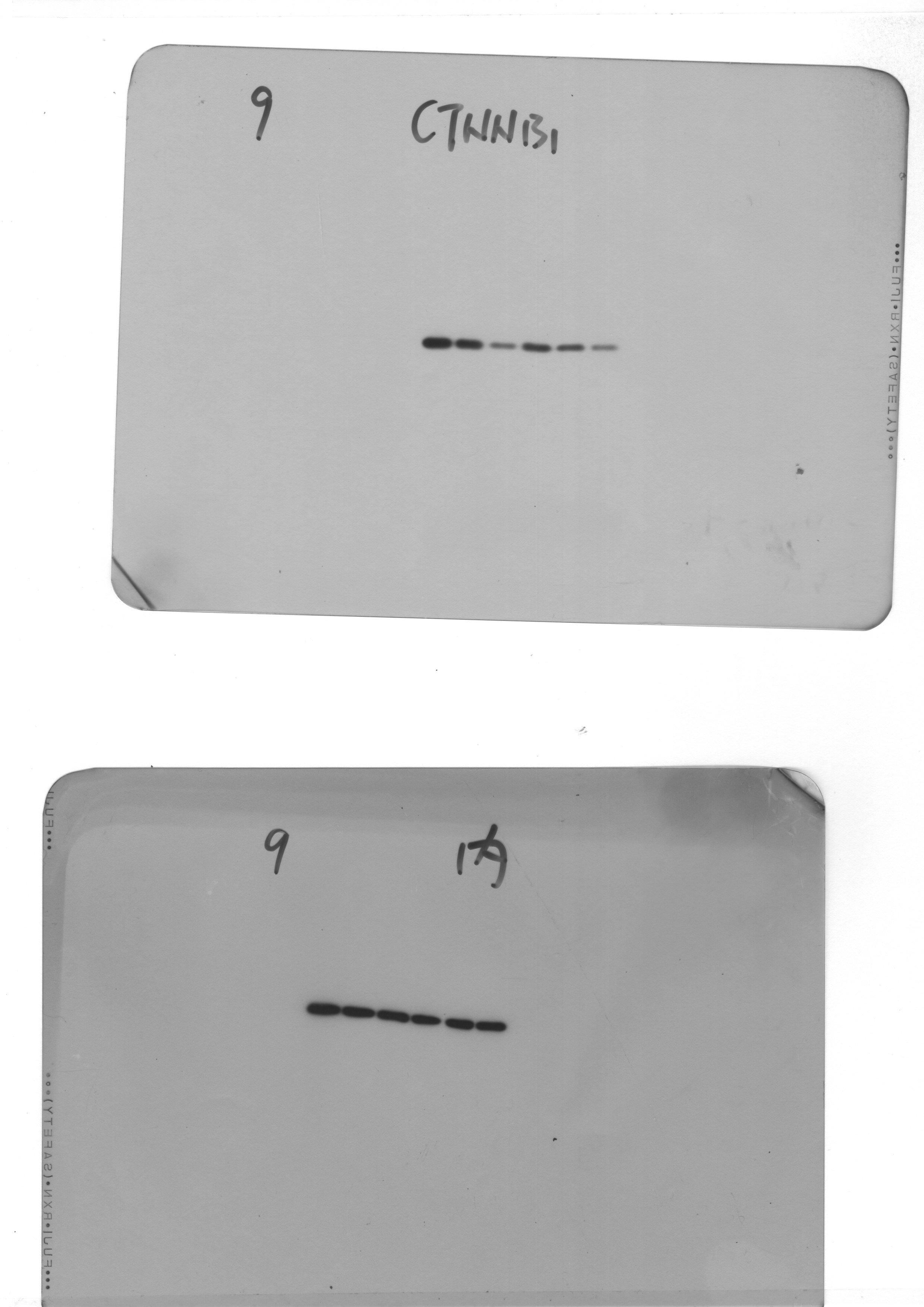

Supplement: Supplementary file 2 [file DataSheet2.ZIP › Experimental data/protein electrophoresis image.tif]
